# Experimental Investigation of Shear Connection in Precast Concrete Sandwich Panels with Reinforcing Ribs

**DOI:** 10.3390/polym18020200

**Published:** 2026-01-11

**Authors:** Jan Macháček, Eliška Kafková, Věra Kabíčková, Tomáš Vlach

**Affiliations:** Faculty of Architectural Engineering, Czech Technical University in Prague, Thakurova 7, 166 29 Prague, Czech Republic; jan.machacek@fsv.cvut.cz (J.M.); eliska.kafkova@fsv.cvut.cz (E.K.); vera.kabickova@fsv.cvut.cz (V.K.)

**Keywords:** high-performance concrete, shear reinforcement, carbon reinforcement, fibre reinforced polymer, precast concrete sandwich panels, rigid heat insulation, reinforcing rib

## Abstract

This paper presents an experimental investigation of the shear connection between outer layers of lightweight precast concrete sandwich panels (PCSP) made of high-performance concrete (HPC). The shear-transfer mechanism is based on reinforcing ribs composed of rigid polymer-based thermal insulation combined with carbon-fibre-reinforced polymer (CFRP) shear reinforcement. A total of seven full-scale sandwich panels were tested in four-point bending. This study compares three types of rigid thermal insulation used in the shear ribs—Purenit, Compacfoam CF400, and Foamglass F—and investigates the influence of the amount of CFRP shear reinforcement on the structural behavior of the panels. Additional specimens were used to evaluate the effect of reinforcing ribs and of polymer-based thermal insulation placed between the ribs. The experimental results show that panels with shear ribs made of Purenit and Compacfoam CF400 achieved significantly higher load-bearing capacities compared to Foamglass F, which proved unsuitable due to its brittle behavior. Increasing the amount of CFRP shear reinforcement increased the load-bearing capacity but had a limited effect on panel stiffness. The experimentally determined composite interaction coefficient ranged around α ≈ 0.03, indicating partial shear interaction between the outer concrete layers. A simplified strut-and-tie model was applied to predict the load-bearing capacity and showed conservative agreement with experimental results. The findings demonstrate that polymer-based materials, particularly CFRP reinforcement combined with rigid polymer insulation, enable efficient shear transfer without thermal bridging, making them suitable for lightweight and thermally efficient precast concrete sandwich panels.

## 1. Introduction

The shear connection between the outer concrete layers has a major influence on the resulting mechanical properties of the panel; therefore, the connection method is a very important part of panel designing. Historically, the shear connection was solved using steel reinforcement [1,2,3,4] or concrete ribs [5]. These solutions have a few problems. The major one is, that these types of solutions cause significant heat bridges and due to this fact, the panels are devaluated from a thermal-technical point of view. Another problem is that steel-based reinforcement is corrosive and requires a relatively wide cover layer which results in wide outer concrete layers and subsequently also heavy sandwich panels.

Nowadays, the problem of shear connection is investigated by many scientists all over the world, for example, Wonchang Choi [6], Ali Shams [7] and Thomas G. Norris [8], and current solutions mostly contain nonmetal shear connectors and, instead of metal-based reinforcement, the polymers are reinforced by carbon, basalt or glass fibres. Nonmetal reinforcement is not corrosive, which allows the use of thin cover layers, and it is a poor heat conductor, so the problem with heat bridges disappears. In any case, reinforcement made of fibre-reinforced polymers is suitable only for transmission of tensile forces, which is a problem because the inner layer is formed of heat insulation, which is usually very soft and absolutely is not able to transfer high compressive loading [9,10]. As a result, the mechanical behavior of polymer-based materials and their interaction with concrete becomes a governing factor for the structural response of modern precast sandwich panels.

From a materials perspective, the structural performance of non-metallic shear connection systems is governed by polymer mechanics and interface behavior. FRP-based connectors are typically efficient in tension but may exhibit brittle failure modes, limited compressive resistance, and bond-slip controlled response depending on the polymer matrix and surface treatment. When rigid polymer-based insulation is used as a structural rib, its compressive and shear response becomes critical for preventing core crushing and controlling interlayer slip. Consequently, the polymer–concrete interface and the stiffness of the polymer insulation directly influence the degree of composite action of precast concrete sandwich panels, which is commonly quantified using composite interaction coefficients. Several recent studies have investigated the shear performance of FRP and polymeric connectors in precast sandwich panels, including parametric experimental investigations and numerical analysis of connector behavior [11,12,13].

For the shear connection to function properly, it is necessary to prevent the middle layer of thermal insulation from being crushed. This problem is often solved by the use of thermal insulation with high compressive strength—for example, compacfoam, lightweight concrete, etc.—throughout the area. These insulations are used alone [14] or in combination with nonmetal reinforcement [15,16]. But this also presents a problem, because they are very expensive or very heavy.

Therefore, the solution presented in this paper consists of reinforcing ribs which are created by a combination of rigid thermal insulation and CFRP reinforcement, which means that panels will not be extremely heavy or expensive and at the same time they will have relatively good shear connection without thermal bridges [17]. The proposed concept intentionally employs polymer-based materials not only for thermal efficiency but also as load-carrying components governing shear transfer and partial composite interaction.

Although recent studies have investigated FRP shear connectors and composite behavior of precast concrete sandwich panels, most available solutions focus on discrete connectors or grid-based systems, while the structural role of rigid polymer-based insulation ribs remains insufficiently explored [18]. In particular, there is a lack of experimental data on full-scale panels where polymer insulation is deliberately used as a shear-carrying web in combination with CFRP reinforcement, and where the resulting partial composite action is quantified. This research addresses this gap through experimental testing of full-scale panels and evaluation of their composite interaction.

This paper contains load test results of seven panels which were tested in a four-point bending test. Firstly, three panels which had different types of thermal insulation used as material of the reinforcing rib were tested. In particular these were purenit, compacfoam CF400 and foamglass F. These materials are commonly used to solve construction and technical issues from a thermal engineering point of view [19,20,21]. The insulations were compared based on the price and mechanical properties of the final panels, and in the following research only the best one was used. Afterwards, the influence of the amount of shear reinforcement on the mechanical properties of the panel were tested. For this research, another two panels were created. In this paper can be found a comparison between three panels with the same thermal insulation used as material for the reinforcing rib but with different amount of shear reinforcement. In the following chapter are shown results which demonstrate the influence of reinforcing ribs and the influence of rigid thermal insulation used between shear ribs on the mechanical properties of the final panel. For this purpose, another two panels were created. The first one was without reinforcing ribs, only with rigid thermal insulation between outer concrete layers, and the second with two reinforcing ribs with rigid thermal insulation between them. As rigid thermal insulation, PIR foam was used. Lastly, this paper contains a presentation of a calculation method which could be used for designing the sandwich panels. Specifically, it is a strut-and-tie model. The calculation method was also compared with real behavior of the panel during the loading test.

## 2. Materials and Methods

### 2.1. Materials

#### 2.1.1. Concrete Mixture

Thin outer layers of precast concrete sandwich panels (PCSP) were made of high-performance concrete (HPC) [22], specifically the mixture whose composition is presented in Table 1. The mixture was originally developed in the Department of Architectural Engineering, CTU in Prague. This concrete is a self-compacting, fine-grained mixture with a maximum aggregate size of 1.2 mm. It was designed for special applications involving very thin structural elements, where high workability, smooth surface quality, and durability are required. Such a composition makes the material particularly suitable for prefabrication of thin HPC elements. Material properties adopted from manufacturer datasheets were used for reference purposes, while the critical mechanical behavior relevant to the panel response was verified experimentally. To determine the basic mechanical properties of concrete, a total of six samples were tested: three cubes with an edge length of 100 mm and three prisms with the dimensions 40 × 40 × 160 mm. The cubes were tested in compression according to EN 12390-3 [23] and the beams were subjected to a three-point bending test according to EN 12390-5 [24].

The compressive strength of the concrete was determined by standard cube tests in accordance with EN 12390-3 [23], yielding an average value of 97.42 MPa. This value represents a basic material parameter and is used in this study solely as an input for the structural analysis; therefore, detailed load–displacement curves are not presented. In addition, the average tensile strength of the concrete under bending was measured on beams at 15.51 MPa according to EN 12390-5 [24]. The modulus of elasticity was 45.00 GPa according to ČSN EN 12390-13 [25].

#### 2.1.2. Carbon-Fibre-Reinforced Polymer Reinforcement

**Figure 1:**
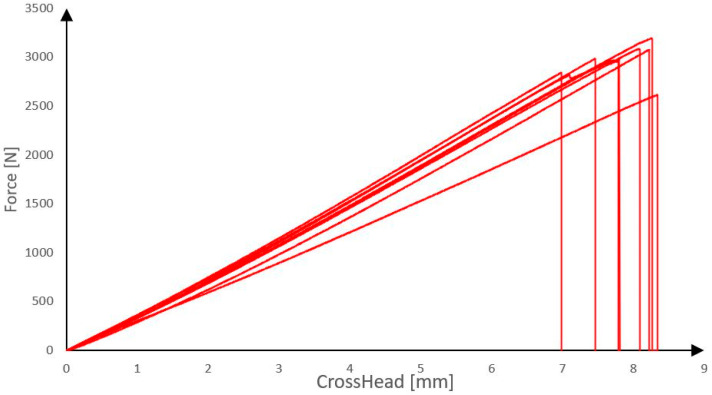
Tensile test of carbon composite reinforcement.

**Figure 2:**
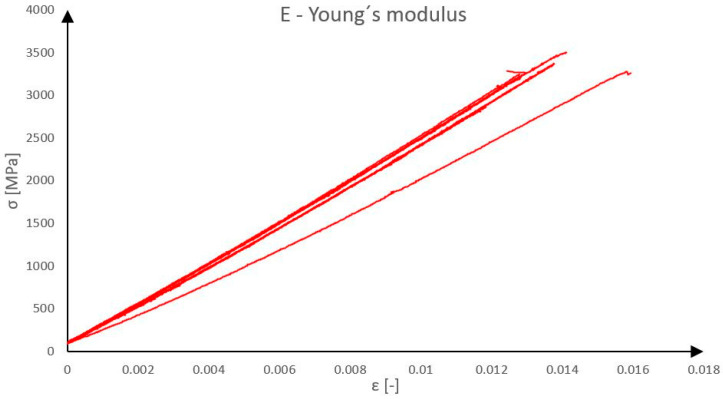
Young’s modulus of carbon composite reinforcement.

As a shear reinforcement of the reinforcing rib, a composite reinforcement was used consisting of epoxy resin and carbon fibres TenaxTH—E STS40 F13 24k 1600 tex from TEIJIN. Before the fabrication of the panels, eight test specimens of the single composite roving reinforcement were first prepared and tested by uniaxial simple tension on the test equipment for determining basic mechanical parameters. The maximum force at material failure was monitored (bearing capacity) and the dependence of the relative deformation on stress (approximate Young’s modulus of elasticity) [22]. The results of the tests are presented in Figure 1 and Figure 2.

The linear density of the carbon rovings (1600 tex) is reported for material identification, while the mechanical behavior is consistently described using force (N) and stress (GPa), which are used as input parameters for the structural analysis. Tensile tests of individual carbon composite reinforcements revealed that the rovings achieved average tensile strength of 2968 N, corresponding to a tensile stress of 3.28 GPa, and an average Young’s modulus of 238 GPa. The measured values of the actual composite reinforcement parameters will serve as input data for the development of a numerical model. For comparison, the theoretical maximum values provided in the manufacturer’s technical datasheet for the roving are a tensile strength of 4000 MPa and an elastic modulus of 240 GPa.

#### 2.1.3. Purenit

Purenit is a construction material based on rigid polyurethane foam (PIR), characterized by excellent thermal insulation, mechanical, and chemical properties [22]. It is therefore often used to eliminate thermal bridges. In this study, Purenit was incorporated into the panel design as a shear web material in combination with braided composite reinforcement. Additionally, below, alternative materials such as rigid polystyrene and foam glass were also evaluated. As part of this investigation, the behavior of Purenit under compressive loading was tested. Three samples of width 100 mm, thickness 30 mm and height 180 mm were examined. The height of 180 mm corresponds to the designed height of the sandwich panel web, which is why this atypical dimension was selected. These dimensions were precisely measured at three locations using a caliper, and the average values were calculated. The samples were then loaded at the rate of 1 mm per minute until the failure.

**Figure 3:**
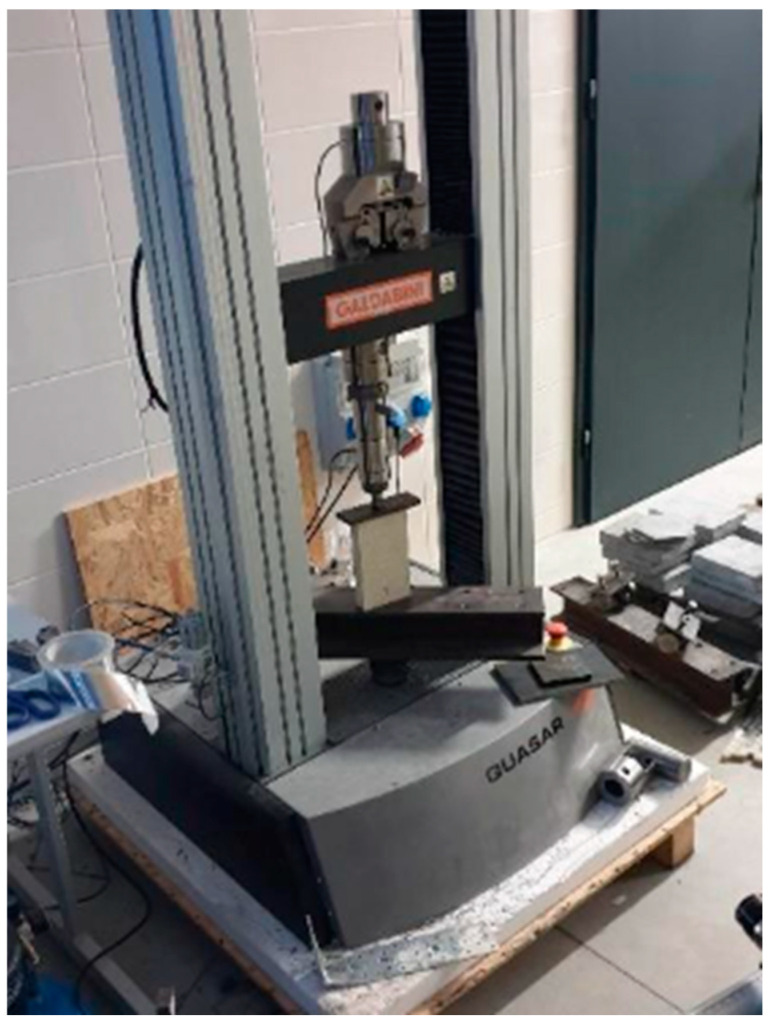
Compress test of Purenit.

The compressive behavior of Purenit was investigated by uniaxial compression tests performed using a testing machine (Galbadini Quasar, Italy) equipped with rigid steel loading plates. The specimens were placed centrally between the plates and loaded under displacement control at a constant rate of 1 mm/min. No lateral confinement was applied, allowing free lateral expansion of the material. The load was introduced through full-area contact between the specimen ends and the steel plates, and the alignment was carefully checked to minimize eccentric loading. The selected specimen height of 180 mm corresponds to the thickness of the shear rib in the sandwich panel and thus captures the stability and progressive crushing behavior relevant for the in-panel application. In the sandwich panel, the rigid insulation core of the reinforcing rib primarily carries the compressive principal stress component within the shear-transfer mechanism, while the CFRP braid resists the tensile principal stresses. Although the stress state in the rib is multi-axial due to combined compression and shear, the uniaxial compression test provides the key material parameters required for simplified modelling, namely the compressive strength and an effective modulus of elasticity in the initial linear range. The same testing procedure and boundary conditions were applied for the other investigated insulation materials, i.e., Compacfoam CF400 and Foamglass F, to ensure a consistent comparison of their suitability for use as rib core materials. A view of the sample during the experiment is presented in Figure 3. From the compression loading tests, the load–displacement diagram of Purenit was obtained.

**Figure 4:**
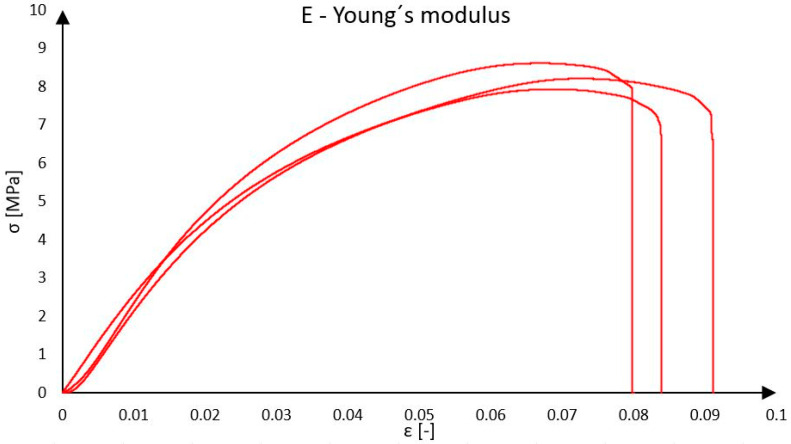
Young’s modulus of Purenit.

Based on the results of compression loading tests, the average compressive strength of Purenit was determined to be 8.25 MPa, as presented in Figure 4, and the modulus of elasticity was 226 MPa, which approximately corresponds to the data provided in the manufacturer’s technical datasheet [26]. After exceeding the load-bearing capacity, the material fractured violently, and the load-carrying capacity dropped abruptly to zero. Up to the stress level of approximately 3.50 MPa, the material’s behavior was elastic. The results of the experimental measurements of mechanical parameters, including stiffness, will also be used in the further calculations.

#### 2.1.4. Compacfoam CF400

**Figure 5:**
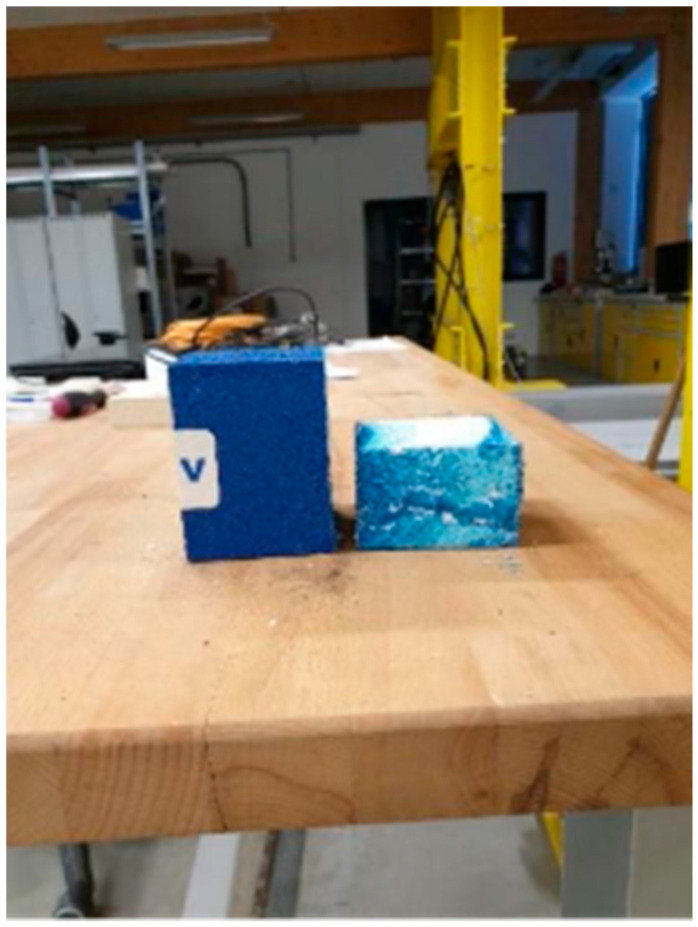
Compacfoam samples before and after loading.

**Figure 6:**
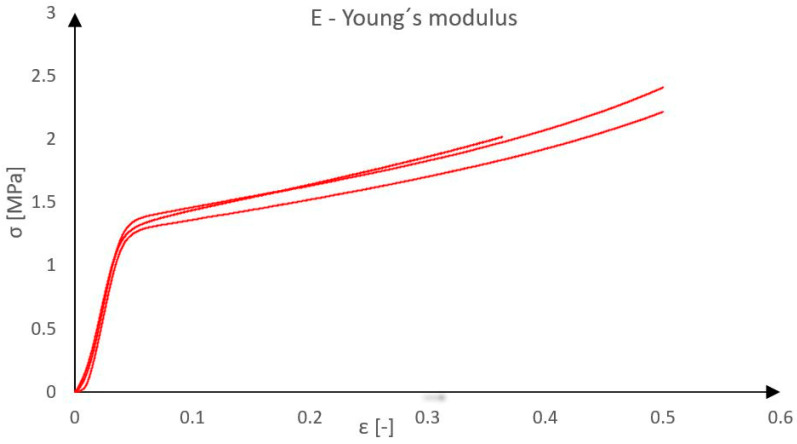
Young’s modulus of Compacfoam CF400.

As a material alternative to Purenit, the material Compacfoam was also evaluated [14]. Compacfoam is a thermoplastic foam based on polystyrene, characterized by high compressive strength and low thermal conductivity, commonly used as a construction insulation material for eliminating thermal bridges. A total of three Compacfoam samples with dimensions of 80 × 75 × 85–100 mm were tested under compressive loading. A view of the sample is presented in Figure 5. Samples’ dimensions were precisely measured at three locations using a caliper, and the average values were calculated. The samples were then gradually loaded at a rate of 1 mm per minute until the moment of failure. From the tests, the load–displacement diagram of Compacfoam CF400 was obtained, as presented in Figure 6.

The material exhibited linear elastic behavior up to the stress level of approximately 1.2 MPa, after which the rate of compression increased. However, no sudden collapse occurred. Essentially, it can be stated that the applied load would continue to increase until the specimen was completely flattened, which is typical behavior for this type of plastic material. Therefore, the ultimate compressive strength cannot be precisely determined, as the force on the testing machine would continue to rise. The average value of the modulus of elasticity in the linear part of the force-displacement diagram was 27.15 MPa.

#### 2.1.5. Foamglass F

**Figure 7:**
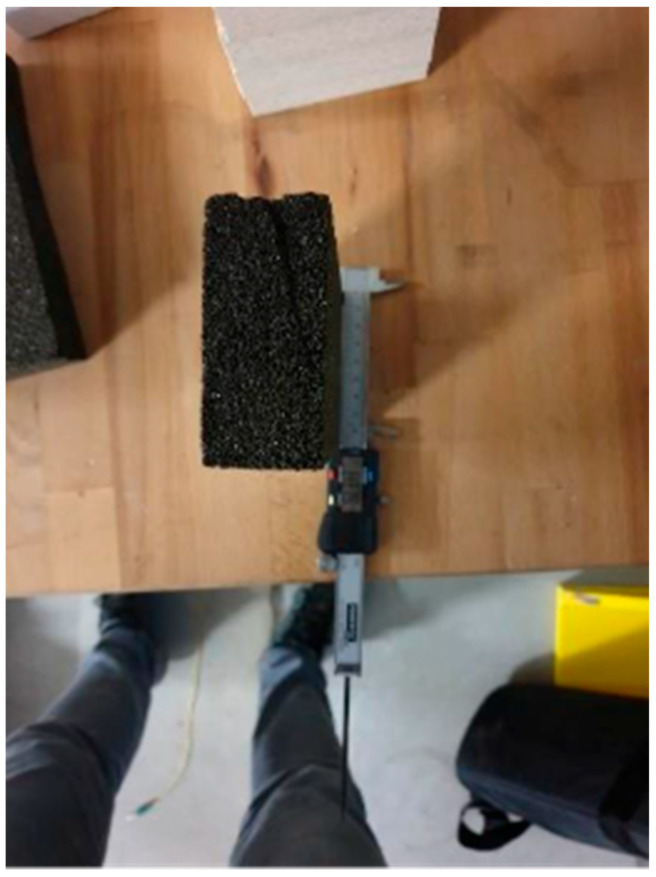
Foamglass—sample measurement.

**Figure 8:**
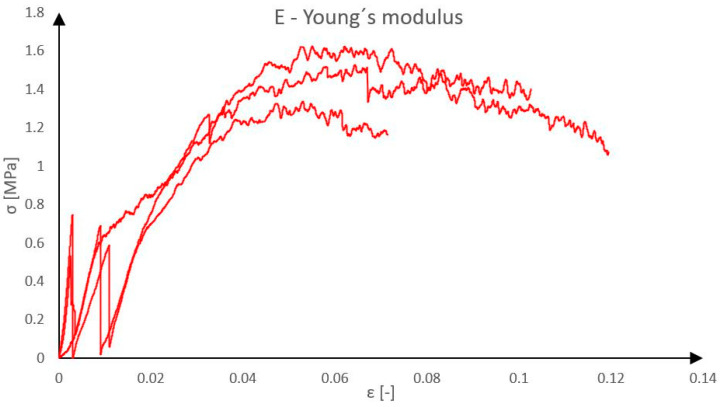
Young’s modulus of Foamglass F.

The last tested material was foamglass, considered as a potential alternative to Purenit [17]. As with other types of rigid thermal insulation, three samples were tested. The specimens were 90 mm wide, 40 mm thick and 180 mm high, corresponding with the thickness in final sandwich panel. A view of the sample is presented in Figure 7. The dimensions were measured at three locations, and the average values were calculated from obtained measurements. The samples were gradually loaded at a rate of 1 mm per minute until the moment of failure. From the loading tests, the force-displacement diagram of Foamglass F was obtained, as shown in Figure 8. In the graph, several initial jumps can be observed, which indicate significant local damage of the test specimen around the steel loading plate, most likely due to improper end finishing of the sample. In this case, it would be better to use a binder, such as gypsum, between the steel plate and the sample.

The behavior of the material was completely different from the previously tested ones, as it is made from recycled glass, which is inherently brittle. The material is highly sensitive to local loading. During testing, it began to crumble progressively under the applied load (collapse of pores rather than of the entire structure) and it can be assumed that if the test had not been stopped, the loading would have continued until the specimen was completely crushed. The average compressive strength was measured to be 1.50 MPa, which approximately corresponds to the manufacturer’s technical data sheet. Due to the material’s behavior under loading, it was difficult to determine the modulus of elasticity. In further calculations, it was processed with a value of 37.17 MPa, which was derived from the linear portion of the graph. It should be noted that the compression test results for Foamglass F were influenced by imperfect end-contact conditions, which led to local damage at the specimen–plate interface and an initial non-smooth load–displacement response. Therefore, the obtained results are considered primarily qualitative and are used to assess the general suitability and failure mode of Foamglass F for shear rib applications rather than to derive precise mechanical parameters.

#### 2.1.6. Basalt Fibre Reinforced Polymer

Basalt fibre reinforcement polymer (BFRP) was employed as the reinforcement of the outer concrete sheets. BFRP was selected due to its high tensile strength, low density, and excellent corrosion resistance, making it a suitable replacement for conventional steel reinforcement in concrete structures. Alternatively, dispersed steel microfibers could also be considered as a suitable reinforcement option; however, this variant was not included in the demonstrative experiment. A grid-type reinforcement produced by ORLITECH company was used, featuring a mesh size of 50 mm and a reinforcement strand diameter of 2.2 mm. The mechanical properties of the material were not determined experimentally. Instead, the values provided in the manufacturer’s technical sheet were adopted for subsequent calculations. According to the manufacturer, the tensile strength of the material is 1215 MPa and Young’s modulus of elasticity 37 GPa [27,28].

### 2.2. Specimen Preparation

#### 2.2.1. Reinforcing Ribs

**Figure 9:**
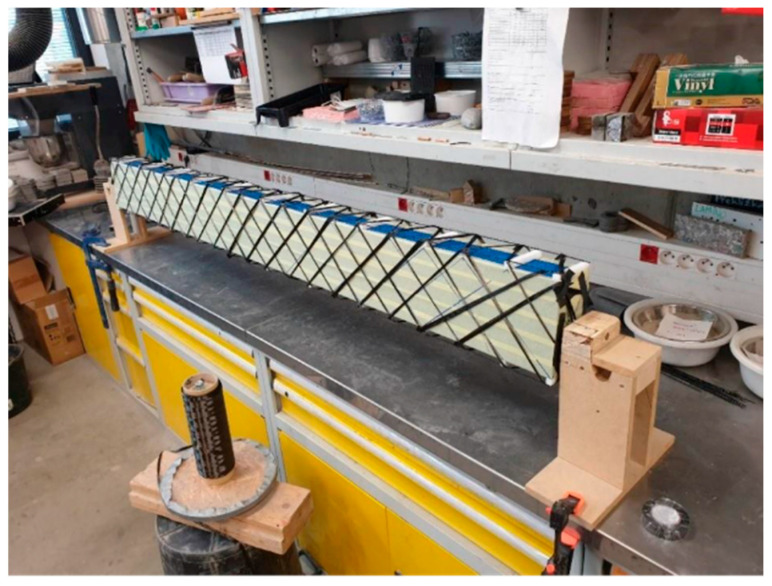
Reinforcing rib preparation.

The first step in the specimen preparation was creation of the reinforcing ribs. Rigid thermal insulation in three variants was cut to the required panel length dimensions. Subsequently, the selected carbon fibres were braided around it. The fibres were placed at the required spacing and oriented approximately at 45° in both directions, forming a sleeve-like knitted layer. It was essential to maintain a gap between the edges of the insulation and the reinforcement to ensure proper anchorage of the shear reinforcement within the outer concrete layers. For this purpose, polystyrene spacers in combination with basalt rovings were used, providing a 1 cm gap. A view of the carbon reinforcement after winding is shown in Figure 9. Carbon fibres were homogenized using epoxy resin. To improve adhesion to the concrete the end, the CFRP single roving shear reinforcement was sanded with fine aggregate, producing a roughened surface [29].

#### 2.2.2. Concreting of the Lower and Upper Layers

**Figure 10:**
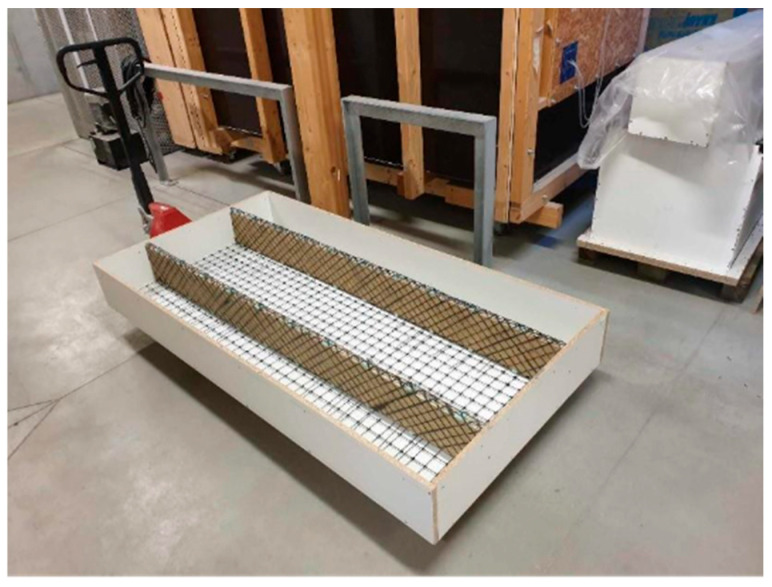
Formwork ready for concreting of the lower layer.

The second step was the casting of the lower concrete layer. For the panel specimens, the thickness of both HPC layers was designed to be 20 mm. Prior to casting, the formwork was assembled, and the shear ribs were placed inside it and fixed in their correct positions using screws anchored into the front face of the mould. In the next step, BFRP for the grid reinforcement was installed. The grid was hooked onto the CFRP loops (acting as spacers) and positioned at the required elevation—approximately at mid-depth of the panel—using additional spacers made from mirelon. A view of the prepared form is presented in Figure 10. The lower layer was then cast without vibration, as the applied mix was self-compacting. During casting, the uniform thickness of the concrete layer across the entire formwork was checked, as well as any potential upward floating of the BFRP reinforcement, which has a lower density than concrete.

**Figure 11:**
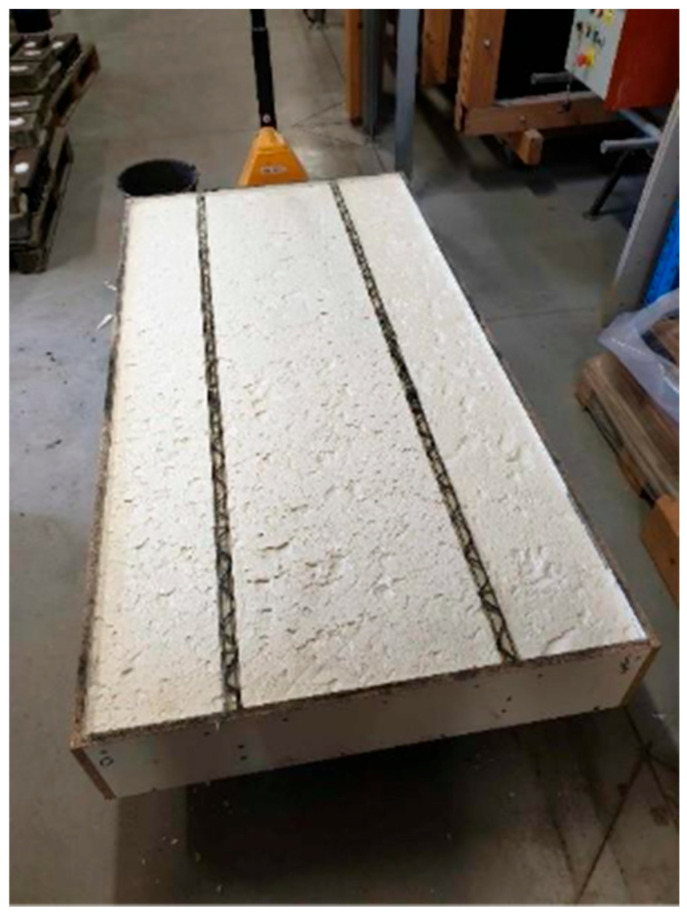
Rigid thermal insulation as lost formwork.

Three days after casting the first layer, wooden spacer blocks were installed, and the formwork for the upper layer was placed on them at the required distance and fixed with screws. For the panel variant with rigid thermal insulation between the ribs, the insulation was inserted directly and used as permanent formwork, without installing an additional temporary layer as seen in Figure 11. Subsequently, the BFRP reinforcement grid was installed in the same manner as for the first layer and hooked onto the CFRP loops from the top side of the ribs, identical to the procedure used for the lower layer. Mirelon spacers were again used to set the correct position, placing the reinforcement approximately at mid-depth of the concrete layer. The upper layer was then cast in the same manner as the lower one. After a further 28 days of curing, the sandwich panel was ready for the loading test.

### 2.3. Calculation Methods and Loading Process

#### 2.3.1. Strut-And-Tie Model

The strut-and-tie model is a simplified method of calculation, where the panel construction is represented by the rib’s longitudinal section. In the following analysis, all materials are assumed to behave in a linear elastic manner up to failure; the CFRP shear reinforcement is considered to carry tensile forces only, perfect bond between polymer-based reinforcement and concrete is assumed, and time-dependent effects such as creep and relaxation of polymer materials are neglected due to the short-term static loading conditions of the experiments. The strut-and-tie model works as follows: the upper compression bar represents the upper outer concrete layer, and the lower tension member represents lower outer concrete layer or its reinforcement, so in the calculation model they are from appropriate profiles with areas corresponding to real parts and with properties which must be measured on materials samples before. The diagonals represent shear reinforcement, so their area and properties correspond to areas and pre-measured properties of the used reinforcement. The last part of the calculating model are shafts, and they represent rigid thermal insulation with its area and pre-measured mechanical properties. The model is loaded by forces applied at positions corresponding to the experimental test which needs to be simulated. The goal is to find the loading force at which the calculated stress in some part of the panel has the breaking strength value. This method is very effective for determination of the load-bearing capacity of the final panel but determination of stiffness of the construction is almost unusable. The main advantage is that this method is simple and does not require expensive computing technology nor complex computing software [19].

#### 2.3.2. Composite Interaction Coefficient

**Figure 12:**
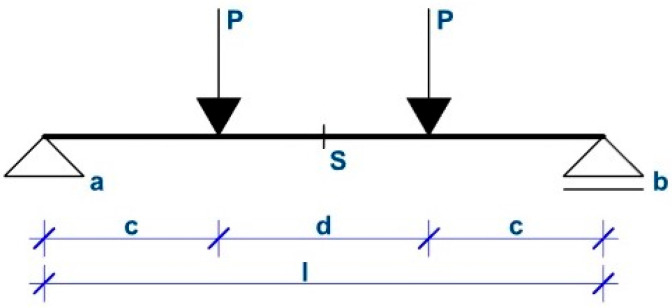
Schema of the four-point bending test [27].

To evaluate the effectiveness of the shear connection between the outer concrete layers, the composite interaction coefficient serves as an appropriate indicator. Once this coefficient is determined experimentally, it can also be used to estimate the actual stiffness of the structure. The calculation of the composite interaction coefficient for a panel tested in a four-point bending test as presented in Figure 12 (which corresponds to the tests performed within the framework of this research) is described by the equations below. Finally, it is assessed whether combining the determination of the panel strength limit using the strut-and-tie model with the evaluation of stiffness based on the composite interaction coefficient provides a reasonably accurate description of the actual structural behavior under loading [15,30].

(1) $$EI=E_{C}\times \frac{b\times {t_{1}}^{3}}{12}+E_{TI\;}\times \frac{b\times {t_{TI}}^{3}}{12}+E_{C}\times \frac{b\times {t_{2}}^{3}}{12}$$

Equation (1). Stiffness of the panel without shear ribs.

(2) $$EI=E_{C}\times \frac{b\times {t_{1}}^{3}}{6}$$

Equation (2). Stiffness of the panel when influence of thermal insulation is neglected, and outer layers are the same width.

(3) $$EI=E_{C}\times \frac{b\times {t_{1}}^{3}}{6}+E_{C}\times \frac{b\times t_{1}\times {\left(t_{1}+t_{TI}\right)}^{2}}{2}$$

Equation (3). Stiffness of the panel while ensuring perfect shear interaction.

(4) $$EI=E_{C}\times \frac{b\times {t_{1}}^{3}}{6}+{\alpha \times E}_{C}\times \frac{b\times t_{1}\times {\left(t_{1}+t_{TI}\right)}^{2}}{2}$$

Equation (4). Stiffness of the panel considering imperfect shear interaction.

(5) $$w=\frac{P\times c}{24\times EI}\times (3\times l^{2}-4\times c^{2})$$

Equation (5). Deformation of the construction in four-point bending test [31].

(6) $$w=\frac{P\times c}{24\times {(E}_{C}\times \frac{b\times {t_{1}}^{3}}{6}+{\alpha \times E}_{C}\times \frac{b\times t_{1}\times {\left(t_{1}+t_{TI}\right)}^{2}}{2})}\times (3\times l^{2}-4\times c^{2})$$

Equation (6). Equation (4) inserted into Equation (5).

(7) $$\alpha =\frac{\frac{P\times c\times (3\times l^{2}-4\times c^{2})}{24\times w}-E_{c}\times \frac{b\times t_{1}^{3}}{6}}{E_{c}\times \frac{b\times t_{1}\times (t_{1}+t_{TI})}{2}}$$

Equation (7). Calculation of the composite interaction coefficient.

EI = Bending stiffness, b = panel width, t_1_ = width of the first outer concrete layer, t_2_ = width of the second outer concrete layer, t_TI_ = width of the thermal insulation, E_c_ = modulus of elasticity of concrete, E_TI_ = modulus of elasticity of thermal insulation, α = composite interaction coefficient, w = deformation, P = force, (c, d, l) = coefficients describing geometry.

#### 2.3.3. Four-Point Bending Test

**Figure 13:**
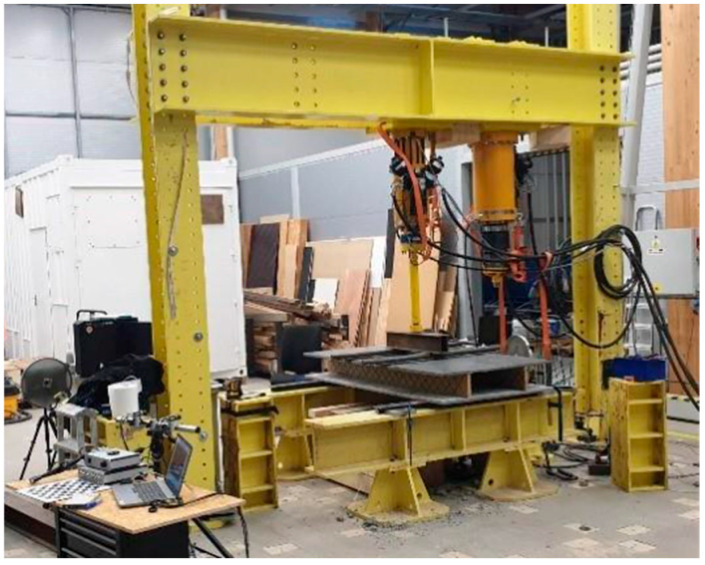
The test assembly.

All the samples were tested in a four-point bending test. The distance between supports was 1.5 m. The load acted exactly in the thirds of the span. The test assembly is displayed in Figure 13. The reinforcing ribs were created from Purenit, Compacfoam CF400 and Foamglass F. Total of three panels were tested, one for each material. The other materials and geometric specifications were same for all panels. Every sample was 2 m long, 1 m wide and 22 cm high. Both outer concrete layers were 2 cm high. For every sample, the same type of HPC and the same type of shear reinforcement were used. Also, the amount of shear reinforcement was the same. CFRP 1600 tex was used, which was applied at an angle of 45°, and the distance between reinforcing rods was 10 cm. The distance between the ribs was 0.5 m. The only difference was in the rigid thermal insulation. The rib’s width design was based on their compressive strength and was performed so that all ribs can carry roughly the same pressure load. All the samples were tested to failure. The loading of the sample was carried out with a constant displacement of 2 mm per minute. The specimen was loaded under a constant displacement rate of 2 mm per minute. The displacement was monitored using potentiometric sensors as well as the DIC method, which was employed to observe the behavior of the rib during loading.

## 3. Results and Discussion

### 3.1. Comparison Between Panels with Reinforcing Ribs Made of Different Types of Rigid Thermal Insulations

**Figure 14:**
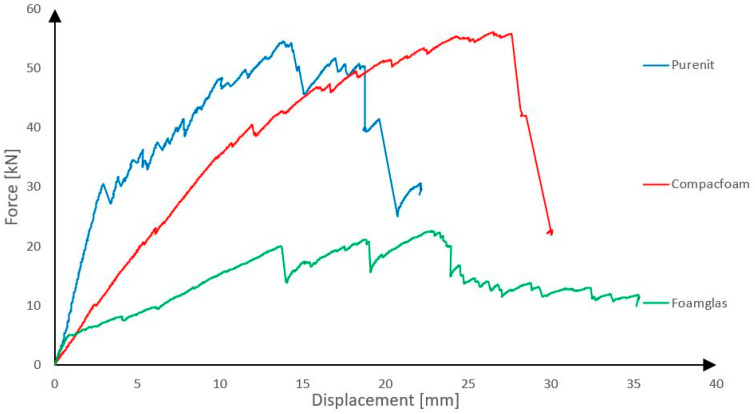
Comparison between panels with different rigid thermal insulations used as reinforcing ribs.

In the following sections, a distinction is made between the initiation of failure, defined as the first rupture of the CFRP shear reinforcement, and the ultimate collapse of the panel, which occurred at higher load levels due to progressive shear failure and crushing of the rib core. The load–displacement curves presented in Figure 14 show the applied force as a function of mid-span displacement measured during the four-point bending test. Three panels were tested in each group, and a representative specimen was selected for the presentation of the results. The panels with three different shear rib materials are shown in the figure.

The observed differences between the panels can be attributed not only to the compressive strength of the insulation materials but also to their stiffness and fracture characteristics. Purenit exhibits a relatively high elastic modulus combined with a limited but stable elastic range, which enables efficient transfer of compressive stresses in the rib while maintaining sufficient deformation capacity prior to failure. In contrast, Foamglass behaves as a highly brittle material, where local crushing and pore collapse initiate early and lead to unstable damage propagation, resulting in reduced load-bearing capacity and limited post-elastic response.

**Figure 15:**
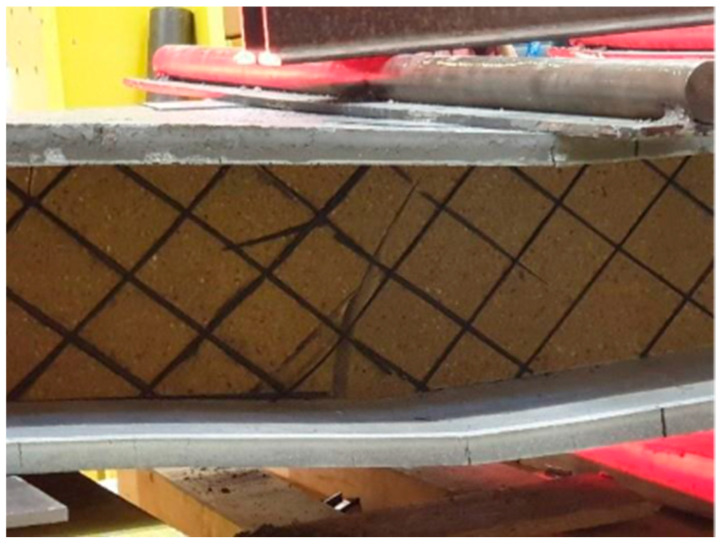
Shear failure of the Purenit rib.

**Figure 16:**
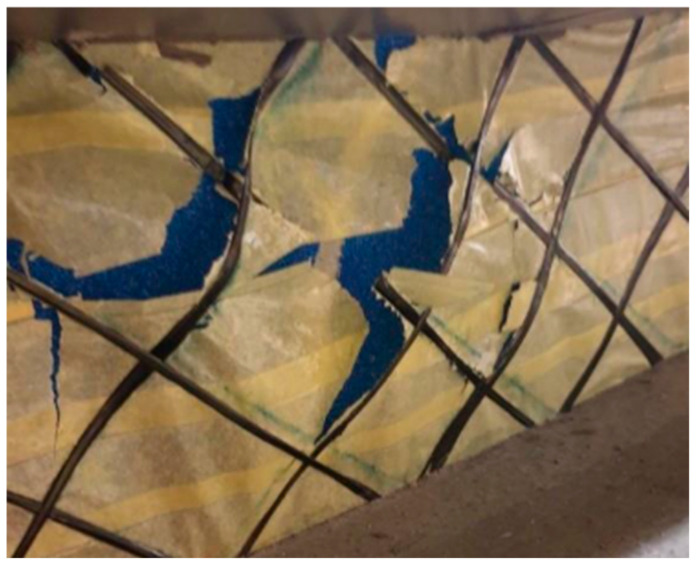
Shear failure of the Compacfoam rib.

It is evident from the graph that the panels with shear ribs made of Compacfoam and Purenit exhibit significantly higher load-bearing capacity, indicating that Foamglass is not suitable for this purpose. The panel with Purenit shear ribs shows nearly the same load-bearing capacity as the Compacfoam panel but demonstrates greater stiffness. In addition, Purenit is a less expensive material than Compacfoam and does not require a separation layer between insulation and epoxy resin used for homogenization of carbon reinforcement roving. Based on these findings, subsequent research focuses exclusively on panels with reinforcing shear ribs made of Purenit. In this series of experiments, all three panel versions failed due to rupture of the shear reinforcement followed by shear failure of the rigid thermal insulation. The observed shear failures are shown in Figure 15 and Figure 16.

### 3.2. Comparison Between Panels with Different Amounts of Shear Reinforcement

**Figure 17:**
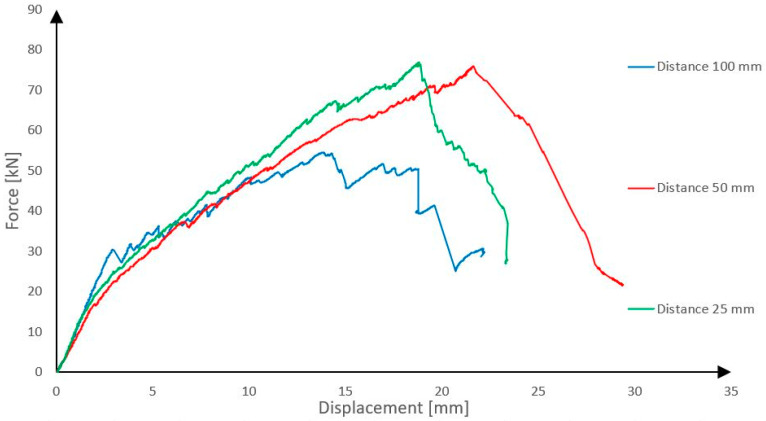
Comparison between panels with different amount of shear reinforcement.

A total of three different sets of specimens were tested. The material and geometric parameters were identical for all panels and correspond to the specifications given in the previous chapter about different ribs. Purenit was used as the material for the rigid shear ribs, as it demonstrated the most favorable performance in the preliminary tests. The only difference between the tested specimens was the amount of shear reinforcement. For all panes, CFRP 1600 tex rovings were used. However, the spacing between the shear-reinforcing single rods was 10 cm in the first configuration, 5 cm in the second and 2.5 cm in the third. All specimens were loaded until failure. The test results and load–displacement curves for representative specimens are presented in Figure 17.

**Figure 18:**
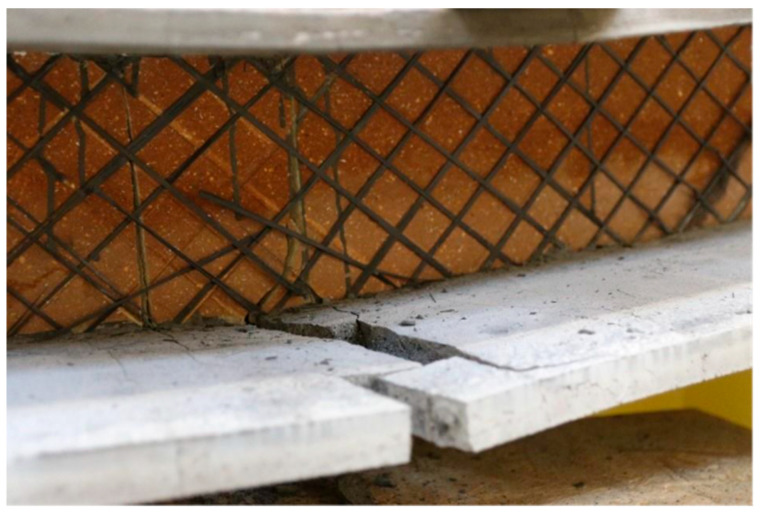
Interruption of the lower concrete layer.

As shown in the graph, the amount of shear reinforcement does not have a significant effect on the stiffness of the panel. However, it does influence its load-bearing capacity. The panel with shear reinforcement spacing 50 mm and 25 mm exhibited similar load-bearing capacities, as both specimens failed due to rupture of the lower concrete layer together with its BFRP reinforcement. In these cases, the shear ribs were therefore not the critical element governing failure. The failure mode is illustrated in Figure 18.

### 3.3. Influence of Reinforcing Ribs and of the Thermal Insulation Used Between Ribs

**Figure 19:**
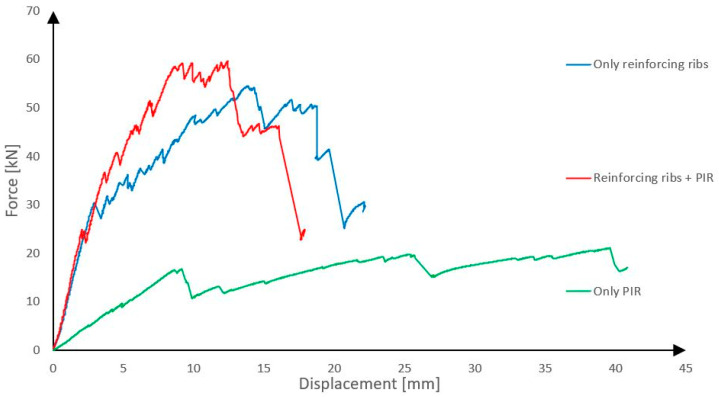
Influence of reinforcing ribs and of the thermal insulation used between ribs.

To demonstrate the influence of reinforcing ribs on the load-bearing capacity and rigidity of the panel, a total of three samples were tested. The material and geometric parameters were the same for each panel and corresponds to the specification stated in the chapter *3.1 Comparison between panels with reinforcing ribs made of different types of rigid thermal insulations*, but one panel was created without reinforcing ribs. In this case, the space between outer layers was filled by PIR-based thermal insulation. The remaining two panels had reinforcing ribs made of Purenit, and one of them had nothing between the ribs, which represents cases when very soft thermal insulation is used between ribs. The last panel had spaces between ribs filled with PIR based thermal insulation. The results are shown in Figure 19.

**Figure 20:**
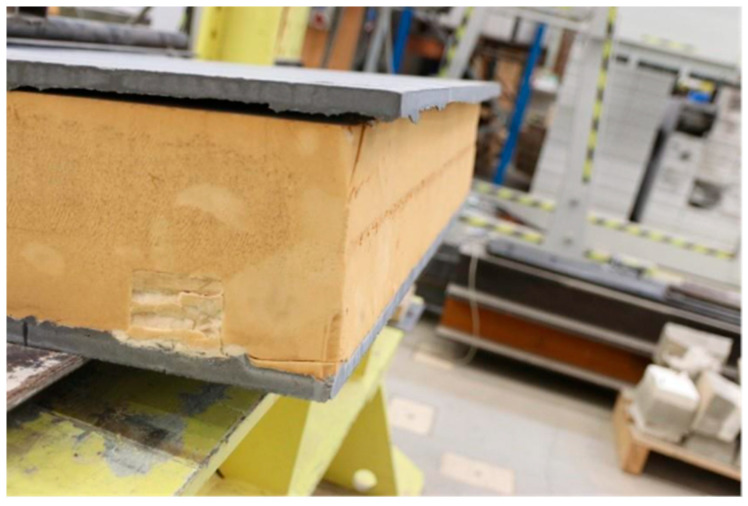
Displacement of the individual layers.

**Figure 21:**
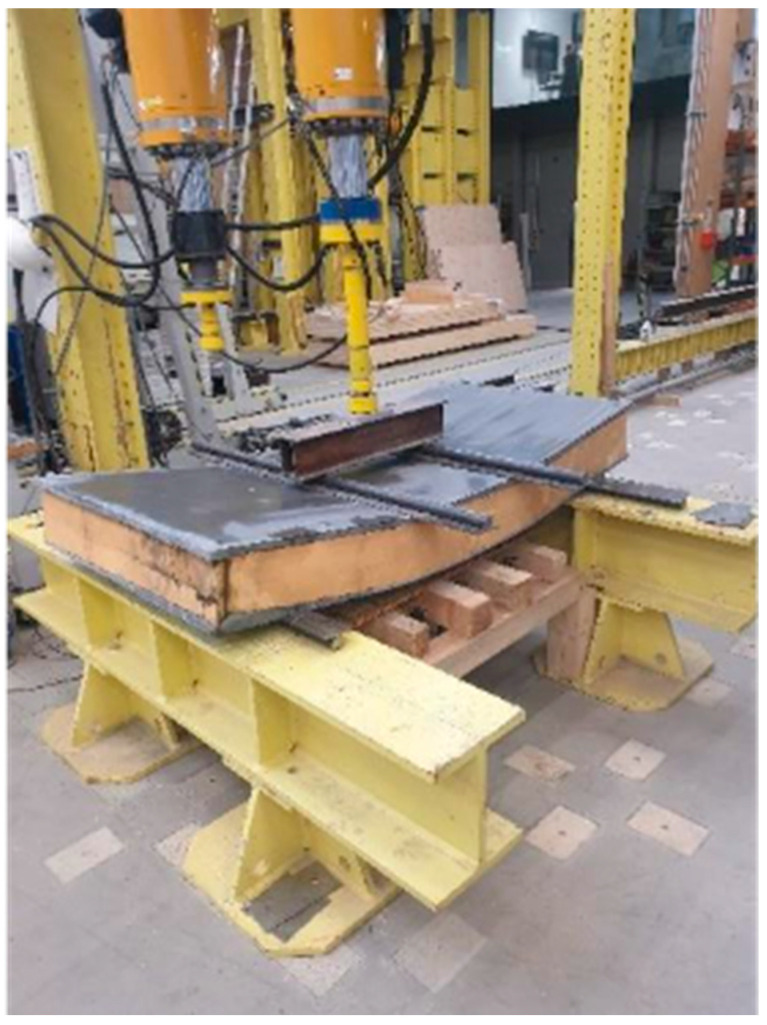
Deformation of the panel without reinforcing ribs.

The graph clearly shows that panels with reinforcing ribs have significantly better properties. Load-bearing capacity is much greater, and panels are more rigid. The graph also shows that PIR used between reinforcing ribs has an influence on the rigidity of the panel in the plastic part of the loading. The impact on the load-bearing capacity is small. When loading a panel without shear ribs, the mutual displacement of the individual layers of the panel was clearly visible. This effect can be observed in Figure 20 and the overall view of the sample after the test is then presented in Figure 21.

### 3.4. Strut-And-Tie Calculation Model

**Figure 22:**
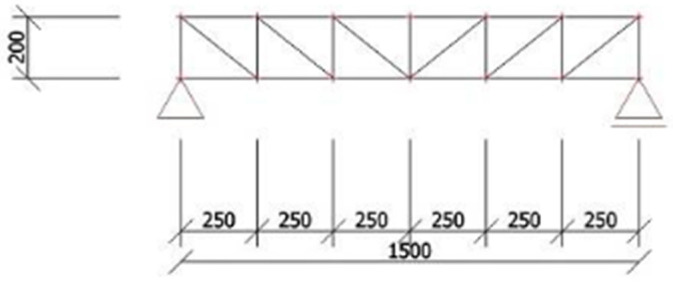
Strut-and tie-model [19].

**Figure 23:**
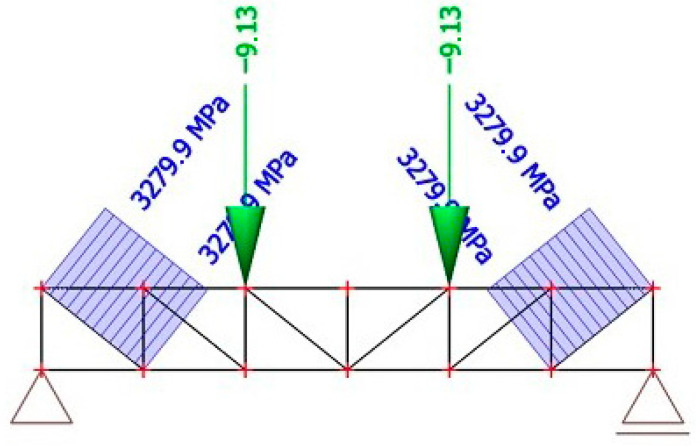
Strut-and tie-model with marked maximum tensile stress value [19].

The model as presented in Figure 22 and Figure 23 has been loading by two forces operating in thirds of the truss beam until the calculated stress in diagonals has the breaking strength value [19]. Figure 22 and Figure 23 schematically illustrate the adopted strut-and-tie idealization of a single reinforcing rib, including the representation of compressive struts, tensile ties, and the applied loading configuration. Compacfoam CF400 shows a markedly different behavior, characterized by lower stiffness but pronounced ductility. This allows progressive deformation and redistribution of stress after the initiation of damage, which explains the comparable ultimate load-bearing capacity but reduced stiffness of the corresponding panels. These results highlight that an optimal rib core material should combine sufficient stiffness to limit interlayer slip with controlled ductility to prevent sudden brittle failure.

Because the strut-and-tie model represents only one rib and the experimental panel has two, the forces must be summarized and multiplied by two. So, the prediction based on this calculation model is that after the force on the press reaches 36.52 kN, the shear reinforcement will break and then the panel collapses due to shear failure of the rib from Purenit.

**Figure 24:**
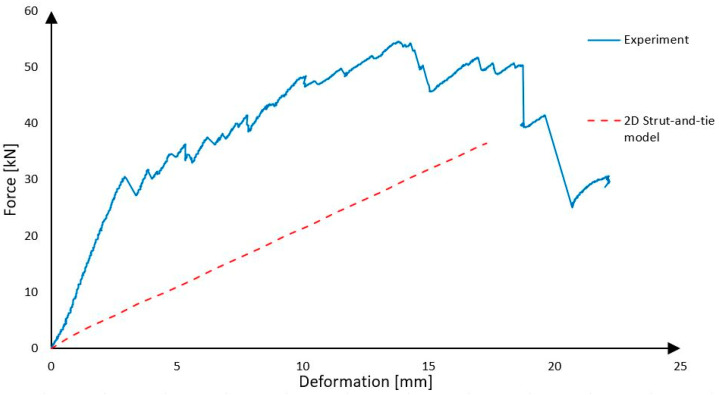
Experiment vs. strut-and-tie model.

As it is presented Figure 24, the strut-and-tie model predicted that the panel will collapse when the force on the press reaches 36.52 kN due to failure of the shear reinforcement. It is very close to the real force, 30.00 kN, at which the shear reinforcement started to fail. The strut-and-tie model predicts the load level corresponding to the initiation of failure, i.e., the rupture of the CFRP shear reinforcement, whereas the experimentally observed ultimate collapse of the panel occurred at significantly higher loads due to the presence of post-elastic load-carrying mechanisms. The panel collapsed when the force on the press was 54.58 kN, which was much more than was expected. The same can be stated for the deformations, which were smaller than expected, because Purenit with a large cross-sectional area also has large effect. It is possible to say that the model provides results which are “on the safe side”, so this model could be used for the approximate design of the precast concrete sandwich panels, because in the construction practice, we mainly design structures so that they move only in the linear part of their working diagram when loaded, and irreversible deformations do not occur. However, the model does not perfectly describe the actual behavior. The plastic part of the loading is not included in the results, and the rigidity of the construction does not correspond to reality [19].

To secure more accurate results, it is possible to combine load-bearing capacity calculated by the strut-and-tie model with the composite interaction coefficient. For all panels with reinforcing ribs made of Purenit, the interaction coefficient was calculated by substituting into Equation (7). The results are shown in Table 2.

P = 8.0 kN, c = 0.5 m, l = 1.5 m, b = 1.0 m, t_1_ = 0.02 m, t_TI_ = 0.18 m, E_c_ = 45.0 GPa, w = data from experiment

**Figure 25:**
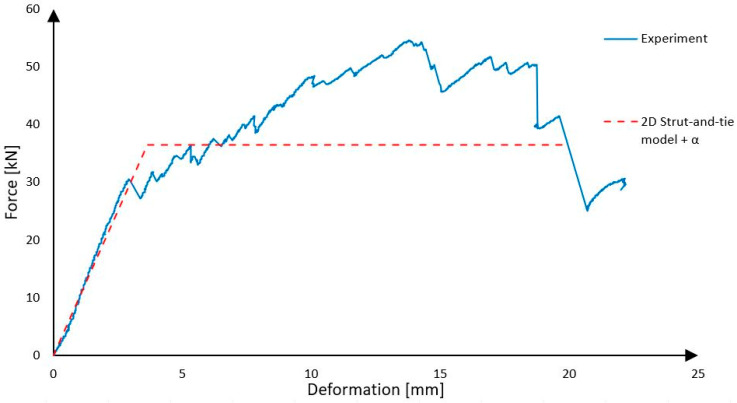
Strut-and-tie model in combination with α.

It is evident from Table 2 that regardless of the amount of carbon braid around the ribs, the value of the composite interaction coefficient is around 0.03. The results of the calculation which combines the strut-and-tie model and average value of α are shown in Figure 25.

As is obvious from the graph, this bilinear diagram describes the behavior of the panel during loading relatively precisely. But the problem is that the value of α was established based on only a few experiments and all samples had the same geometry. So, for this type of calculation, it is necessary to do many experiments and create a database of data from the tests. But in the case of a panel with the same geometry as was tested, we can simply predict that α is going to have a value around 0.03 and that the amount of reinforcement will influence only load-bearing capacity. Whether this α value also applies to panels with different geometries is a question for further research.

Durability-related testing was not included in the study, as the primary focus was on the short-term structural behavior and shear-transfer mechanism of the proposed system. The use of HPC for the outer layers and CFRP reinforcement is, however, expected to provide enhanced durability compared to conventional reinforced concrete systems, due to reduced permeability of HPC and the corrosion resistance of CFRP materials. The absence of steel shear connectors further limits the risk of corrosion-related degradation.

## 4. Conclusions

The reinforcing ribs increase the load bearing capacity and rigidity of the precast concrete sandwich panels. Purenit is the most appropriate thermal insulation for the shear rib purpose in comparison with Foamglass and Compacfoam, but there are many other interesting materials which could be tested in the future. The amount of the shear reinforcement made of CFRP does not have a significant impact on rigidity of the panel, but it increases the panel’s load-bearing capacity. To improve the rigidity of the panels, it is possible to use some type of rigid thermal insulation between reinforcing ribs. To predict the load-bearing capacity of the panel, the strut-and-tie model could be used, and it provides relatively precise results; however, it cannot predict deformation during loading. For this purpose, it might be possible to calculate with the coefficient of composite interaction, but this thesis must be verified by further research. Further research of precast concrete sandwich panels does not have to focus only on shear connection between outer concrete layers; there are many other challenging topics which should be solved, for example, fire resistance of the panels, connection between adjacent panels, connection between panels and supporting structure, acoustic parameters, etc. In the end, it is important to mention that due to lack of funds, only one sample of each type of panel was created and tested, which means that repeating the same experiments may not yield the same results.

## Figures and Tables

**Table 1:** HPC mix design.

Mix Content	kg/m^3^
Cement I 42.5R	650
Technical silica sand	1200
Elkem microsilica 940 U-S	100
Technical quartz powder ST 6	235
Superplasticizer based on PCE	18
Water	190
Total	2393

**Table 2:** Composite interaction coefficient.

Type of Panel	α [-]	W [mm]	F [kN]
Shear reinforcement $\dot{a}$ 100 mm	0.0317	1.52	16
Shear reinforcement $\dot{a}$ 50 mm	0.0258	1.83	16
Shear reinforcement $\dot{a}$ 25 mm	0.0295	1.62	16
Shear reinforcement $\dot{a}$ 100 mm + PIR	0.0361	1.35	16

## Data Availability

The original contributions presented in this study are included in the article. Further inquiries can be directed to the corresponding author.
